# A Novel Synonymous Mutation of SARS-CoV-2: Is This Possible to Affect Their Antigenicity and Immunogenicity?

**DOI:** 10.3390/vaccines8020220

**Published:** 2020-05-14

**Authors:** Sung-Jae Kim, Van-Giap Nguyen, Yong-Ho Park, Bong-Kyun Park, Hee-Chun Chung

**Affiliations:** 1 Department of Veterinary Medicine Virology Lab, College of Veterinary Medicine and Research Institute for Veterinary Science, Seoul National University, Seoul 08826, Korea; kvirus7734@gmail.com (S.-J.K.); parkx026@snu.ac.kr (B.-K.P.); 2 Department of Veterinary Microbiology and Infectious Diseases, Faculty of Veterinary Medicine, Vietnam National University of Agriculture, Hanoi 100000, Vietnam; nguyengiap83@gmail.com; 3 Department of Veterinary Microbiology, College of Veterinary Medicine and Research Institute for Veterinary Science, Seoul National University, Seoul 151-742, Korea

**Keywords:** COVID-19, SARS-CoV-2, spike protein, antigenicity

## Abstract

The S glycoprotein of coronaviruses is important for viral entry and pathogenesis with most variable sequences. Therefore, we analyzed the S gene sequences of SARS-CoV-2 to better understand the antigenicity and immunogenicity of this virus in this study. In phylogenetic analysis, two subtypes (SARS-CoV-2a and -b) were confirmed within SARS-CoV-2 strains. These two subtypes were divided by a novel synonymous mutation of D614G. This may play a crucial role in the evolution of SARS-CoV-2 to evade the host immune system. The region containing this mutation point was confirmed as a B-cell epitope located in the S1 domain, and SARS-CoV-2b strains exhibited severe reduced antigenic indexes compared to SARS-CoV-2a in this area. This may allow these two subtypes to have different antigenicity. If the two subtypes have different serological characteristics, a vaccine for both subtypes will be more effective to prevent COVID-19. Thus, further study is urgently required to confirm the antigenicity of these two subtypes.

Coronavirus (CoV) is a class of genetically diverse RNA viruses found in a wide range of hosts including reptiles, birds, and mammals. Most pathogenic CoVs usually cause respiratory and intestinal symptoms in animals [1,2,3,4,5]. Over the past 20 years, a few novel beta-coronaviruses originated from bats have been transmitted to humans and caused severe respiratory syndrome. SARS-CoV and MERS-CoV were first introduced to humans in 2002 and 2012, respectively [3]. Recently, a new novel beta-coronavirus named SARS-CoV-2 first broke out in Wuhan city, China. The first case of this virus was reported in December 2019 and this virus has subsequently spread explosively worldwide and severely threatened human health [3,6].

CoV is generally composed of four major structural proteins: nucleocapsid protein (N), membrane (M), envelope (E), and spike glycoprotein (S). Among these proteins, the S glycoprotein plays crucial roles in viral entry and pathogenesis as its widely exposed structure forms large petal-shaped spikes on the surface of the virion [7]. Mutations in the spike glycoprotein can allow novel coronavirus strains to infect humans and spread pandemically [8]. Therefore, S gene encoding S glycoprotein has widely been used for molecular analysis of coronaviruses due to the significant features of the S glycoprotein affecting the antigenicity and immunogenicity [2,4,9,10,11,12,13]. 

Thus far, there has been little data comparing and analyzing S gene sequences within SARS-CoV-2. Generally, several types of coronavirus are divided into subtypes depending on amino acid mutations in S gene sequences, and molecular analysis based on the S gene can provide insights into antigenicity, immunogenicity, or evolutionary trends [2,4,10,12,13]. Thus, we analyzed the S gene sequences of SARS-CoV-2 to better understand this virus in this study. 

For phylogenetic analysis based on the S gene, 144 sequences of SARS-CoV-2 that globally originated from several countries (China, USA, Italy, Spain, Japan, Vietnam, Taiwan, and Pakistan) were retrieved from GenBank. Using IQ-TREE v1.6.12 [14], the genetic relationships between SARS-CoV-2 were inferred by the maximum likelihood (ML) method. The “-m MFP” option was invoked to help select the data best-fit amino acid substitution model. The branch support values were estimated by ultrafast bootstrap approximation [15] implemented in IQ-TREE [14] via the “-bb 1000” option. The reconstructed phylogenies were displayed and midpoint rooted by FigTree v1.4.3. In the ML tree, completely divided clades were identified among the analyzed SARS-CoV-2 strains (Figure 1A). 

Interestingly, only one reliable synonymous change was found to distinguish between subtypes A and B in this study. SARS-Cov-2a and -2b strains consistently exhibited Ala (D) and Gly (G) at the amino acid sequence position 614, respectively (Figure 1B). The virus consistently evolves to evade the host immune system with synonymous mutations that are so-called positive selection. More evolved viruses are better able to survive, thus such viruses will likely be dominant within the group [16]. SARS-CoV-2a includes the China strains confirmed in 2019, but SARS-CoV-2b only includes the USA strains confirmed after 2020. In addition, the USA was one of the latest countries to experience a COVID-19 outbreak. Although there was only a few months’ difference, SARS-CoV-2b may be a more evolved form. If the mutation of D614G plays a crucial role in the positive selection process, SARS-CoV-2b will be the dominant type of SARS-CoV-2 in the future. More long-term tracking will be required to validate this assumption.

The S glycoprotein is important for viral entry and pathogenesis with the most variable sequences in the coronavirus genomes. Human beta-coronavirus S proteins are cleaved into S1 and S2 subunits by host proteases [17]. The S1 subunit forming a globular shape is responsible for receptor binding [18] while the S2 subunit forming a rod shape mediates membrane fusion [19]. More specifically, the S1 subunit is composed of two major domains (S1-NTD and S1-CTD) and two sub-domains (SD-1 and SD-2). One or both of the major domains is potentially responsible for binding host-receptors, and the sub-domains that are complex folding of elements may allow receptor-induced conformational changes [11,20,21]. Thus, mutations within the S1 region are associated with changes in antigenicity and viral pathogenicity [22]. 

In fact, the S1 subunit contains numerous major and minor neutralizing antibody epitopes [21,23]; thus, it is difficult to investigate all putative epitopes to discover which can play a crucial role on the antigenicity and immunogenicity of viruses. In this situation, inferences considering both aspects of virus evolution and epitope analysis can be helpful to investigate which epitopes really play a crucial role. B-cell epitopes on the S1 subunit were predicted by BepiPred-2.0 [24], the Chou & Fasman method [25], the Kolaskar and Tongaonkar method [26] and Parker’s Hydrophilicity [27]. Regions between amino acids 614 and 621 were equally identified as a B cell epitope by all four methods (Figure 1C). The predicted B-cell epitope including amino acid 614 was located in a relatively well-exposed part of the S1 subunit in the 3D-view structure (Figure 1D); this B-cell epitope sequence was identified within the sequence corresponding to the SD-1/-2 domain (Figure 1E). 

The antigenic indexes of each amino acid in this region (amino acids 613–621) were calculated by the Jameson–Wolf method. When an antigen index was >0.5, it was believed to be a reliable position as an epitope [28]. The results of the antigenic index analysis showed severely reduced indexes of amino acids 615–617 in the SARS-CoV-2b strains compared to SARS-CoV-2a; it is predicted that the change of D614G affects the antigenicity of this region (Figure 1F). Since no amino acid changes were found in this area other than the change of D614G, it is believed that this amino acid change alters the conformation of these immunogenic determinants; consequently, this region is expected to no longer act as a B-cell epitope in SARS-CoV-2b. B-cell plays a major role in recognizing pathogens and stimulating adaptive immunity in the immune response against virus infection. Thus, the elimination of B cell epitopes will likely reduce immunogenicity by hampering the immune cell recognition of the virus [29,30]. 

When reflecting the above results, SARS-CoV-2b may have reduced immunogenicity compared to SARS-CoV-2a. If so, it can permit persistent or recurrent infection of SARS-CoV-2b while evading immune cell recognition. In addition, the mutation of the S1 domain may induce different antigenicity and viral pathogenicity between the two subtypes. Different virus subtypes will likely have somewhat different serological features depending on their antigenicity, although there may be some cross-reactivity. In addition, a certain subtype can serologically cover other serotypes [12,31]. Thus, this point should be considered to create a new SARS-CoV-2 vaccine. Indeed, if the two serotypes have different serological characteristics, a vaccine that includes both subtypes will be more effective at preventing COVID-19, particularly when developing a killed vaccine that has a narrow protection range compared to a live vaccine. This study was confined to investigating the phylogenetic and genetic features of SARS-CoV-2 due to limited information. Therefore, further study on the cross-reactivity between these two subtypes is required to validate our assumption. Viruses have continually evolved through genetic mutations to evade host immune systems in the long history of the fight between humans and viruses. SARS-CoV-2, which has only recently been introduced in humans, will continue to evolve for survival in the current situation, in which this virus has already become a pandemic. To respond properly against this virus, continuous surveillance of this virus’ adaptation to evade host immune systems is important in the future. 

## Figures and Tables

**Figure 1:**
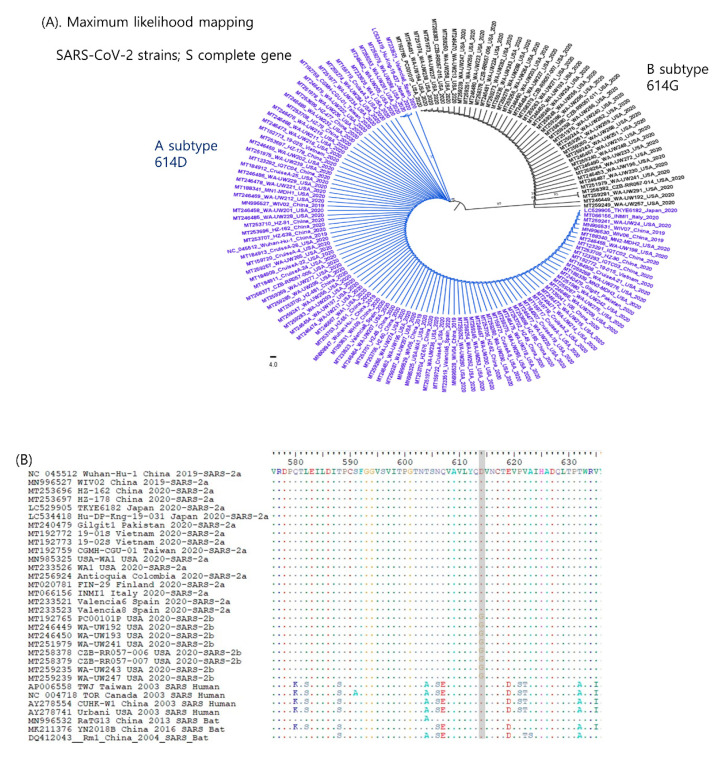

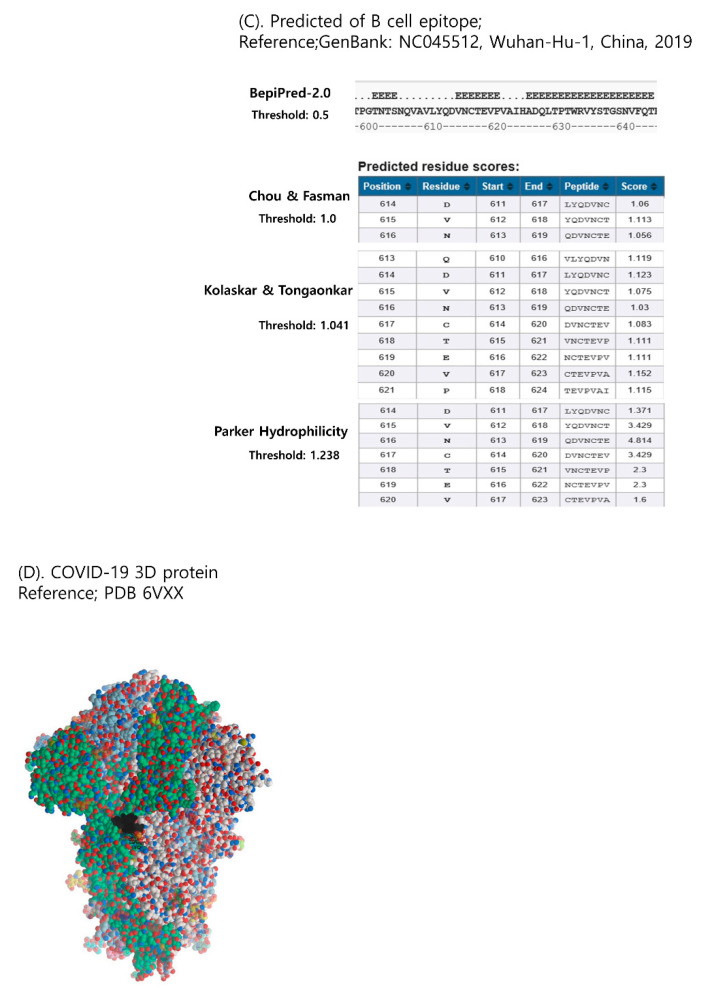

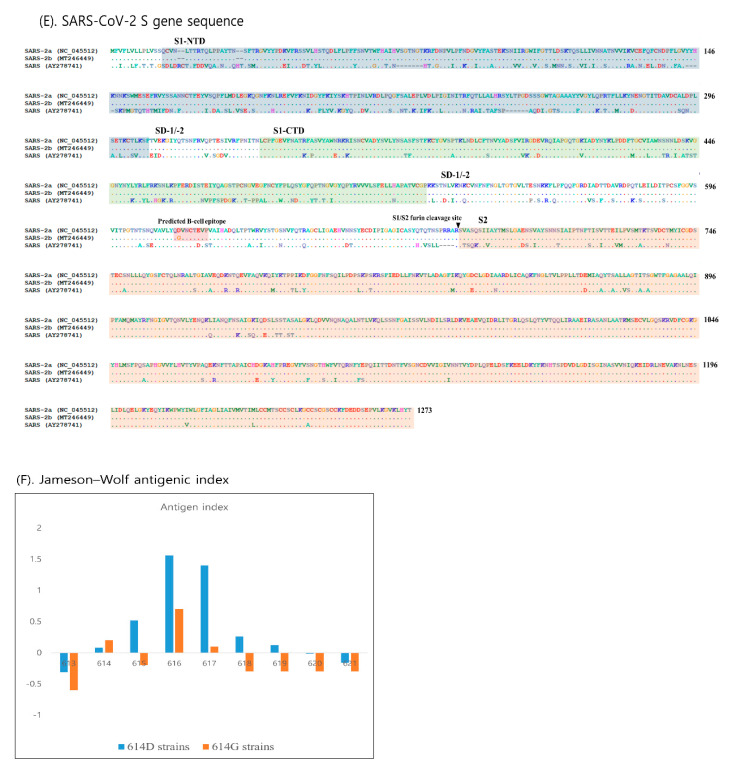
Characterization of the complete S gene in SARS-CoV-2. (**A**) Phylogenetic analysis of SARS-CoV-2 strains based on the S gene. The phylogenetic trees were reconstructed from 144 sequences of SARS-CoV-2 collected globally. Thus, the two subtypes (SARS-CoV-2a and -2b) were completely divided. (**B**) Alignment of SARS-CoV sequences including the aa 614 position are highlighted in gray. A novel reliable synonymous mutation was identified to distinguish the A and B subtypes. SARS-Cov-2a and -2b strains consistently exhibited Ala (D) and Gly (G) at the amino acid sequence position 614, respectively. (**C**) Identification of B-cell epitopes in the adjacent area with aa 614. The B-cell epitope was predicted by BepiPred-2.0 [24], the Chou & Fasman method [25], the Kolaskar and Tongaonkar method [26], and Parker’s Hydrophilicity [27]. The 614–621 region was predicted to consist of epitopes. (**D**) The 3D-structure of SARS-CoV-2 spike protein by Mol soft Mol Browser 3.8–5 according to the original publication from the National Center for Biotechnology Information (NCBI): PDB;6VXX. The predicted B-cell epitope (aa 613–620) highlighted in black color was located at a relatively well-exposed part. (**E**) Sequence alignment of SARS-CoV-1 and -2. The S1 subunit is responsible for receptor binding and the S2 subunit mediates membrane fusion. The S1 subunit consists of two major domains capable of binding to host receptors: an amino (N)-terminal domain (NTD) and a carboxy (C)-terminal domain (CTD) and two sub-domains that may allow receptor-induced conformational changes: SD-1 and SD-2. (**F**) The antigenic index of each amino acid constituting this region (amino acids 613–621) by the Jameson–Wolf method [28].

## Data Availability

The full-length genomes of SARS-CoV 2 strains used are registered within GenBank.
